# Paeoniflorin Induces ER Stress-Mediated Apoptotic Cell Death by Generating Nox4-Derived ROS under Radiation in Gastric Cancer

**DOI:** 10.3390/nu15245092

**Published:** 2023-12-13

**Authors:** Tae Woo Kim

**Affiliations:** Department of Biopharmaceutical Engineering, Dongguk University-WISE, Gyeongju 38066, Republic of Korea; tae1410@naver.com or tae1410@dongguk.ac.kr

**Keywords:** gastric cancer, paeoniflorin, radiation, ER stress, ROS

## Abstract

Gastric cancer is one of the most prevalent cancer types worldwide, and its resistance to cancer therapies, such as chemotherapy and radiotherapy, has made treating it a major challenge. Paeoniflorin (PF) is one potential pharmacological treatment derived from paeony root. However, in cancer, the molecular mechanisms and biological functions of PF are still unclear. In the present study, we found that PF exerts anti-tumor effects in vivo and in vitro and induces apoptotic cell death through ER stress, calcium (Ca^2+^), and reactive oxygen species (ROS) release in gastric cancer cells. However, ROS inhibition by DPI and NAC blocks cell death and the PERK signaling pathway via the reduction of Nox4. Moreover, PF triggers a synergistic inhibitory effect of the epithelial-mesenchymal transition (EMT) process under radiation exposure in radiation-resistant gastric cancer cells. These findings indicate that PF-induced Ca^2+^ and ROS release overcomes radioresistance via ER stress and induces cell death under radiation in gastric cancer cells. Therefore, PF, in combination with radiation, may be a powerful strategy for gastric cancer therapy.

## 1. Introduction

Worldwide, gastric cancer is one of the most common cancer types, and it is the fifth leading cause of cancer deaths globally [1,2]. Gastric cancer therapies include various therapeutic strategies, including chemotherapy, radiation therapy, surgery, immunotherapy, and targeted therapy [3,4]. The most common first-line therapy for gastric cancer is chemotherapy [5,6]. Cytotoxic drugs, such as taxanes, platinum, and fluoropyrimidine, have been used as a first-line therapy for gastric cancer [7,8]. Moreover, perioperative chemotherapy after surgery is a standard and potential therapy for localized resectable gastric cancer [9]. However, perioperative chemotherapy has serious obstacles, including toxicity, adverse effects, and chemo-resistance [10,11]. To solve these obstacles, plant-based natural products, hybrids, and derivatives have been identified as powerful therapeutic strategies for cancer therapy by overcoming chemoresistance and radioresistance [12]. In addition, the combination of anti-cancer drugs and natural products has given rise to a novel field of cancer therapies [13]. Thus, natural products and their derivatives may play potential roles in the development of new drugs and pharmaceutical strategies against cancer and immune diseases.

The extracts derived from the dried root of *Radix Paeoniae Alba* are one of the most generally and frequently used herbal medicines in East Asia [14]. Many reports have indicated that *Radix Paeoniae Alba* extracts exert various pharmacological effects, including anti-cancer, anti-angiogenesis, anti-diabetes, neuroprotection, and anti-inflammation [15,16,17,18,19].

Recent studies have reported that paeoniflorin (PF) derived from *Radix Paeoniae Alba* exerts powerful immune regulatory effects on various autoimmune diseases, including psoriasis, rheumatoid arthritis (RA), Sjogren’s syndrome, and oral lichen planus [20]. PF treatments exert anti-inflammatory effects by inhibiting Cox-2, IL-6, TNF-α, and inducible nitric oxide synthase (iNOS) in immune-regulatory models using lipopolysaccharides (LPS)-mediated Raw264.7 cells [21]. RA is a well-known chronic immune disease, and PF is a novel candidate and strategy to treat RA by alleviating inflammation and physical pain [22]. In a system network analysis using the Kyoto encyclopedia of genes, genome functional analysis, and gene ontology using RA models, it was found that PF targeted eight genes, including IL-6, Fos, LGALS3, TNF, VEGFA, IL-1β, TP53, and STAT3, and PF down-regulated the expression of STAT3, LGALS3, and VEGFA [23]. A recent report has shown that P-glycoprotein (P-gp) is associated with multidrug resistance (MDR) in cancer, and PF treatments down-regulated the expression of P-gp in MDR cell models [24,25]. In addition, PF treatment exerts powerful anti-cancer effects via the upregulation of CDH2, the downregulation of CDH1, and the inhibition of the NF-kB signaling pathway in ovarian cancer cells and cisplatin-resistant ovarian cancer cells [26]. PF inhibits the expression levels of IL-6, IL-1β, TNFα, and the NF-kB signaling pathway in high glucose-induced HUVEC cells [27]. Thus, PF may have powerful immunomodulatory and anti-cancer effects in cancer and inflammatory diseases.

The endoplasmic reticulum (ER) contributes to different cellular functions and processes, including homeostasis, lipid synthesis, protein folding, Ca^2+^ release and storage, and glycogen degradation [28]. However, damaged ER by various stresses, including starvation, hypoxia, Ca^2+^ and ATP depletion, imbalanced pH, and oxidative insults, can cause problems in the functions of ER homeostasis [29]. Prolonged and excessive ER stress induces the unfolded protein response (UPR) and activates signal transduction pathways to restore ER homeostasis [30]. The ER membrane has three sensors: protein kinase RNA(PKR)-like ER kinase (PERK), activating transcription factor 6 (ATF6), and inositol requiring enzyme 1α (IRE1α). These sensors interact with 78 kDa-glucose-related protein (GRP78), also named binding immunoglobulin protein (Bip), on the ER [31]. After the activation of ER stress, PERK is auto-phosphorylated according to the dissociation of GRP78 and PERK, and then the phosphorylated PERK phosphorylates eIF2α in the cytosol. The phosphorylation of eIF2α activates ATF4 and is then translocated into the nucleus [32,33]. The binding of nuclear ATF4 to the CHOP promoter mediates ER-stress-induced apoptotic cell death [31]. Thapsigargin (TG) is an inhibitor of sarco/endoplasmic reticulum calcium ATPase (SERCA) and an inducer of the ER stress response by disturbing intracellular Ca^2+^ stores [32]. The accumulation of unfolded proteins causes apoptosis and cell death via the induction of ER sensors such as PERK, ATF6, IRE1α, and ER stress-induced pathways when the damaged ER cannot be restored [32,33]. The PERK-eIF2α-ATF4-CHOP signaling pathway induces apoptotic cell death and proliferation arrest in various diseases [34]. CHOP induces apoptotic cell death by regulating several pro-apoptotic Bcl-2 family proteins, such as Bcl-2, Puma, and Noxa [35]. Under the ER stress response, CHOP induces apoptotic cell death by down-regulating Bcl-2 and up-regulating Puma and Noxa [36]. Furthermore, the increase in intracellular reactive oxygen species (ROS) and Ca^2+^ release from the ER under ER stress contributes to apoptotic cell death to overcome resistance [37,38,39]. Therefore, PF may be involved in ER stress-related pathways against cancer.

This study aimed to identify a therapeutic strategy to overcome radioresistance through the induction of the PERK-ATF4-CHOP axis in PF-mediated gastric cancer cells and radio-resistant gastric cancer cells.

## 2. Materials and Methods

### 2.1. Reagents

Paeoniflorin (PF, P0038), PERK inhibitor I (GSK2606414; 516535), N-acetylcysteine (NAC), PERK inhibitor II (GSK2656157; 504651), Z-VAD-FMK, lipopolysaccharide (LPS; L4391), diphenyleneiodonium (DPI), and thapsigargin (TG; Millipore, Bedford, MA, USA; T9033) were purchased from Sigma-Aldrich (St. Louis, MO, USA).

### 2.2. Cell Culture

Raw264.7 cells, a macrophage cell type, were purchased from the American Type Culture Collection (ATCC; Rockville, MD, USA) and cultured in Dulbecco Modified Eagle Medium (DMEM; Gibco, NY, USA) with 10% Fetal Bovine Serum (FBS; HyClone, Logan, UT, USA) and a streptomycin-penicillin solution (100 μg/mL streptomycin and 100 I.U./mL penicillin; Gibco-BRL, Gaithersburg, MD, USA). Human gastric cancer cells (NCI-N87, SNU-638, MKN-7, MKN-74, SNU-216, and AGS) were received and purchased from the Korean Cell Line Bank (Cancer Research Center, Seoul National University, Seoul, Republic of Korea). Human gastric cancer cells were cultured and maintained in Rosewell Park Memorial Institute 1640 (RPMI1640) and DMEM mediums (Welgene, Daegu, Republic of Korea) containing 10% inactivated FBS (HyClone, Logan, UT, USA) and a streptomycin-penicillin solution (100 μg/mL streptomycin and 100 I.U./mL penicillin; Gibco-BRL). All the cells were incubated at 37 °C in a 5% CO_2_ incubator.

### 2.3. Cytokine Measurement

Raw264.7 cells (1 × 10^4^ cells/well) were seeded in a 96-well plate with a growth medium. To analyze the cytokines from the cell-cultured medium from the PF-induced Raw264.7 cells, enzyme-linked immunosorbent assays (ELISAs) were performed. The expression levels of TNF-α, IL-6, and IL-1β in the Raw264.7 cells were monitored and analyzed using TNF-α (DY-410; R&D Systems), IL-6 (DY-406; R&D Systems), and IL-1β (DY-401; R&D Systems) ELISA kits. These assays were performed following the manufacturer’s instructions.

### 2.4. Cell Viability and Proliferation Assay

The analysis of the anti-cell viability effects of PF at various times and concentrations on gastric cancer cells was performed by a WST-1 assay (Roche Applied Science, Indianapolis, IN, USA). Gastric cancer cells (1 × 10^4^ cells/well) were seeded and cultured in a 96-well plate. The absorbance of each sample was analyzed at 450 nm using a microplate reader (Molecular Devices, Silicon Valley, CA, USA). These assays were performed according to the manufacturer’s instructions.

### 2.5. LDH Cytotoxicity Assay

The analysis of the LDH cytotoxicity effects of PF at various times and concentrations on gastric cancer cells was performed by an LDH assay (Thermo Scientific, Waltham, MA, USA). Gastric cancer cells (1 × 10^4^ cells/well) were seeded and cultured in a 96-well plate containing cell culture medium. These assays were performed following the manufacturer’s instructions. The absorbance of each sample was analyzed at 490 nm using a microplate reader (Molecular Devices, CA, USA).

### 2.6. Caspase-3 Colorimetric Activity Assay

The analysis of the caspase-3 activity of PF at various times on the gastric cancer cell lines AGS and SNU-638 was conducted by a caspase-3 colorimetric activity assay (Abcam, Milpitas, CA, USA). AGS and SNU-638 cells (1 × 10^4^ cells/well) were seeded and cultured in 96-well plates containing cell culture medium. These assays were performed following the manufacturer’s instructions. The absorbance of each sample was analyzed at 405 nm using a microplate reader (Molecular Devices, CA, USA).

### 2.7. Radiation Exposure

Gastric cancer cells were irradiated at doses of 2, 4, and 6 Gy using a from ^137^Cs source (Atomic Energy of Canada, Ltd., Mississauga, ON, Canada).

### 2.8. The generation of Radioresistance via Irradiation of Parental Cells

Parental AGS and SNU-638 cells seeded and cultured in 60-mm dishes at about 50~60% seeding were treated to 2 Gy for 90 treatment cycles (total 120 Gy) until radioresistance cells were developed. Radio-resistant AGSR and SNU-638R gastric cancer cells were developed.

### 2.9. Colony Formation Assay

The development of radio-resistant in AGS (AGSR) and SNU-638 (SNU-638R) cells was monitored using survival fraction data obtained from a colony formation assay. AGSR, SNU-638R, AGS, and SNU-638 cells were seeded and cultured in 60-mm dishes at a density of 1000 cells/dish and incubated for 24 h before radiation exposure. The colonies were fixed and stained using a 1% methylene blue staining solution. The survival fraction was calculated by the following formula: surviving fraction = number of colonies formed/number of cells seeded * plating efficiency of the control group.

### 2.10. RNA Interference for Knockdown Experiments

AGS and SNU-638 cells were seeded into 6-well plates (4 × 10^5^ cells/well) and incubated overnight with growth media. After incubation, these cells were washed with PBS and incubated for four hours with serum-free media. The knockdown studies used four RNA interference methods (double-stranded siRNAs; 30 nmol/mL, Santa Cruz) to target PERK (Bioneer; 1649-1), CHOP (Santa Cruz; sc-36213), Nox4 (Santacruz), and GRP78 (Santa Cruz; sc-29338) using Lipofectamine 2000 (Invitrogen, Carlsbad, CA, USA) following the manufacturer’s manual.

### 2.11. Quantitative Real-Time Polymerase Chain Reaction

Total RNA was extracted from the cultured cells (2 × 10^6^ cells/well) using Trizol RNA isolation reagents following the manufacturer’s instructions (Invitrogen, Carlsbad, CA, USA). cDNA synthesis was performed using 10 µg of total RNA with a reverse transcription kit (power cDNA synthesis kit; iNTRON, Seongnam, Republic of Korea). The quantitative real-time PCR was performed using ABI Power SYBR green PCR Master Mix (Applied Biosystems, Foster City, CA, USA) following the supplier’s instruction. The primers used were: vimentin (F) 5′-CCAGGCAAAGCAGGAGTC-3′, vimentin (R) 5′-CGAAGGTGACGAGCCATT-3′ (antisense); N-cadherin (F) 5′-GGCATACACCATGCCATCTT-3′, N-cadherin (R) 5′-GTGCATGAAGGACAGCCTCT-3′; E-cadherin (F) 5′-GAACGCATTGCCACATACAC-3′, E-cadherin (R) 5′-GAATTCGGGCTTGTTGTCAT-3′; IL-6 (F) 5′- CTGATGCTGGTGACAACCAC-3′, IL-6 (R) 5′-TCCACGATTTCCCAGAGAAC-3′; IL-1β (F) 5′- GAGTGTGGATCCCAAGCAAT-3′, IL-1β (R) 5′- CTTGTGCTCTGCTTGTGAGG-3′; and TNF-α (F), 5′-ACGGCATGGATCTCAAAGAC-3′, TNF-α (R), 5′-TGAGATAGCAAATCGGCTGAC-3′. The PCR was performed using a Roche Light Cycler 96 System (Roche, Mannheim, Germany). The fold changes of the target genes were normalized to β-actin (F) 5′-AAGGCCAACCGCGAGAAGAT-3′, β-actin (R) 5′-TGATGACCTGGCCGTCAGG-3′, and the relative mRNA expression levels were calculated using the 2^−ΔΔCt^ method.

### 2.12. Western Blotting Analyses

Gastric cancer cells (2 × 10^6^ cells/well) were solubilized in radioimmunoprecipitation assay lysis buffer (RIPA; Biosesang, Inc., Seoul, Republic of Korea) with a protease inhibitor cocktail (Sigma-Aldrich, St. Louis, MO, USA). The total protein was quantified using a BCA protein assay kit (Thermo Scientific, CA, USA). Total protein (20 μg) from AGS and SNU-638 cells was separated using an 8–15% SDS-PAGE gel and then transferred onto a nitrocellulose (NC) membrane (Millipore Corporation, Billerica, MA, USA). The following primary antibodies (1:1000) were used: eIF2α (Santa Cruz; sc-133132), β-actin (Santa Cruz; sc-47778), Nox4 (Proteintech; 14347-1-AP), CD63 (Abcam; ab216130), p-eIF2α (Cell signaling; #3398), CHOP (Cell signaling; #2895), p-P ERK (Cell signaling; #12185), GRP78 (Cell signaling; #3177), cleaved caspase-3 (Cell signaling; #9661), PERK (Cell signaling; #5683), ATF4 (Cell signaling; #11815), and cleaved caspase-9 (Cell signaling; #20750). The secondary antibody used was a horseradish peroxidase-conjugated antibody (Santa Cruz, 1:6000; sc-2357, sc-358914). The membranes were analyzed using a D-Plus enhanced chemiluminescence reaction Pico System (DonginLS, Korea, ECL-PS100) according to the manufacturer’s manual.

### 2.13. Total Exosomes Isolation from Cell Culture Media

AGS and SNU-638 cells were seeded into 100 mm cell culture dishes (2 × 10^6^ cells/well) and incubated overnight with cell culture media. Total exosomes were collected from the cell culture media from the PF (20 μM)-induced gastric cancer cells following the supplier’s instructions (Total Exosome Isolation Reagent, Thermo Scientific, CA, USA).

### 2.14. Intracellular Ca^2+^ Assay

The analysis of the intracellular Ca^2+^ activity of PF at various times on AGS and SNU-638 cells was performed using a caspase-3 activity assay (Abcam, Cambridge, MA, USA). AGS and SNU-638 cells (1 × 10^4^ cells/well) were plated and cultured in a 96-well plate with a growth medium and incubated for 24 h. The cells were treated with PF for 24 h, and a Ca^2+^ activity assay (Abcam, Ca^2+^ Assay Kit [Colorimetric]) was conducted according to the manufacturer’s instructions.

### 2.15. ROS Detection Cell-Based Assay

Cells (1 × 10^4^ cells/well) were seeded and cultured in a 96-well plate containing cell culture media for 24 h, and then these cells were treated with PF. The cells were incubated using the cell-permeant 2′7′-dichlorodihydrofluorescein diacetate (CM-H_2_DCFDA, Invitrogen) for 30 min at 37 °C according to the manufacturer’s instructions. The fluorescence was measured at 495 nm (Ex)/525 nm (Em) using a microplate reader (Molecular Devices, USA).

### 2.16. Animal Experiments for Tumor Xenograft Mouse Models

For the animal study, five-week-old female, athymic BALB/c nude mice (*nu*/*nu*) were purchased from Jung-Ang Lab Animal, Inc. (Seoul, Republic of Korea) and housed for one week with free access to sterile standard mouse chow (Rodent NIH-07 open formula) and water. The animal experiment procedures for tumor xenograft mouse models were performed following the National Institutes of Health guidelines, and the protocol was approved by the Institutional Animal Care and Use Committee of Kyung Hee University (KHSASP-20-250). The mice were randomly divided into three groups (control and PF (250 and 500 mg/kg); *n* = 10 per group). AGS cells (1 × 10^7^) were injected intraperitoneally (ip) into the right flanks of the mice every other day. Tumor volume was calculated on two axes (*L*, longest axis; *W*, shortest axis) three times per week using the formula: (*L* * *W*^2^)/2.

### 2.17. Statistical Analysis

Experimental data are presented as the mean ± standard deviation (SD) of three independent experiments. Statistical analyses were calculated using a Student’s *t*-test. A *p*-value less than 0.05 indicated a significant difference.

## 3. Results

### 3.1. PF Decreases LPS-Induced Pro-Inflammatory Cytokines in Raw264.7 Cells

**Figure 1:**
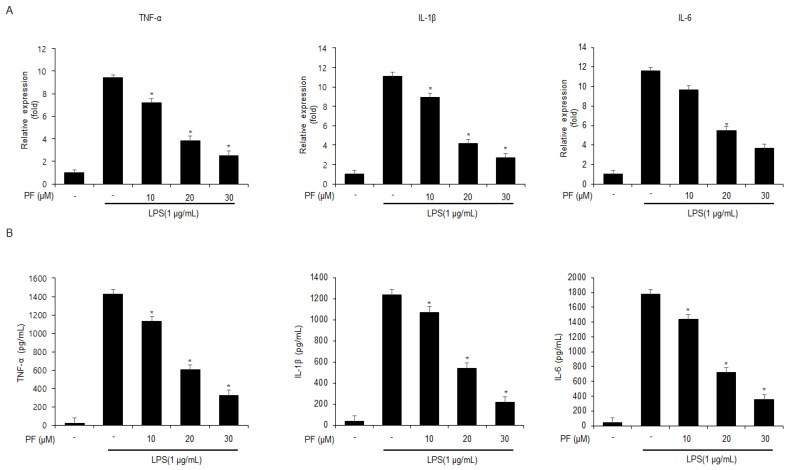
**The effects of PF on TNF-α, IL-6, and IL-1β mRNA and protein expressions in LPS-treated Raw264.7 cells.** (**A**) mRNA levels of TNF-α, IL-6, and IL-1β in LPS (1 µg/mL) -treated Raw264.7 cells with and without PF (0, 10, 20, and 30 µM; 24 h) were measured using a real-time PCR. β-actin was used for normalizing the relative mRNA levels. (**B**) TNF-α, IL-6, and IL-1β protein levels were measured using ELISA assays (* = *p* < 0.05, n.s = no significant). These experiments were repeated three times.

To evaluate if PF can regulate inflammation in LPS-mediated mouse macrophage-like Raw264.7 cells, real-time PCR and ELISA assays were performed. First, the mRNA expression levels of the cytokines IL-6, IL-1β, and TNF-α were enhanced in LPS-treated mouse macrophage-like Raw264.7 cells. Additionally, after LPS pre-treatment, the mRNA expression levels of IL-6, IL-1β, and TNF-α were reduced in the PF-treated mouse macrophage-like Raw264.7 cells (0, 10, 20, and 30 µM; 24 h) (Figure 1A). Next, to test further if PF modulates the levels of IL-6, IL-1β, and TNF-α in LPS-treated mouse macrophage-like Raw264.7 cells, ELISA assays were performed. After LPS pre-treatment, the PF (0, 10, 20, and 30 µM; 24 h) treatment dramatically decreased the release of IL-6, IL-1β, and TNF-α in the PF-treated mouse macrophage-like Raw264.7 cells (Figure 1B).

### 3.2. PF Mediates Apoptosis and Cell Death in Gastric Cancer Cells

**Figure 2:**
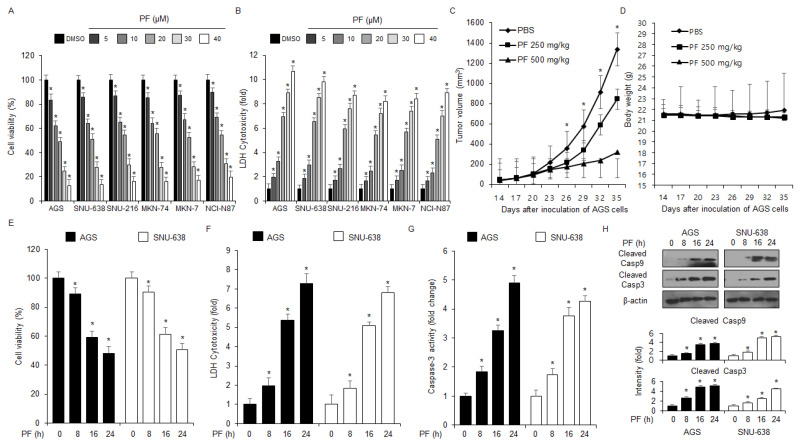
**Anti-gastric tumor effects of PF in vivo and in vitro.** (**A**,**B**) Cell viability and LDH cytotoxicity effects of PF at the indicated concentrations (0, 5, 10, 20, 30, and 40 µM; 24 h) on the gastric cancer cell lines SNU-216, SNU-638, NCI-N87, AGS, MKN-7, and MKN-74, were measured using LDH cytotoxicity and WST-1 assays. DMSO-induced gastric cancer cell lines were set at 100% (* = *p* < 0.05, n.s = no significant). These experiments were repeated three times. (**C**,**D**) Xenograft nude mice were randomly divided into three groups (PBS, PF 250, and PF 500 mg/kg; *n* = 10/group) and implanted (sc) with 1 × 10^7^ AGS cells. PF was administered (ip) once a day for two days. The body weights of the xenograft mice were measured twice a week. (**E**–**H**) The LDH and caspase-3 activities and cell viability of the PF (20 µM)-treated gastric cancer cell lines at the indicated times (0, 8, 16, and 24 h) were measured using LDH cytotoxicity, caspase-3 activity, and WST-1 assays (* = *p* < 0.05, n.s = no significant). These experiments were repeated three times. Western blot assay with total protein from the PF-induced AGS and SNU-638 cell lines was used to measure the protein expression levels of caspase-9 and caspase-3 cleavage in a time-dependent manner. These experiments were repeated three times.

To identify the anti-cancer effect of PF, first investigated the anti-proliferative and cytotoxicity effects of PF on the human gastric cancer cell lines SNU-216, SNU-638, NCI-N87, AGS, MKN-7, and MKN-74, at various PF concentrations (0, 5, 10, 20, 30, and 40 µM; 24 h) were investigated using WST-1 and lactate dehydrogenase (LDH) assays. PF reduced the cell viability and enhanced LDH cytotoxicity in SNU-216, SNU-638, NCI-N87, AGS, MKN-7, and MKN-74 cells at the indicated concentrations (0, 5, 10, 20, 30, and 40 µM; 24 h) (Figure 2A,B). To test the anti-tumor efficacy of PF in vivo, a gastric cancer xenograft mouse model was established using AGS cells. The xenograft mice in the 250 mg/kg and 500 mg/kg PF groups had lower tumor volumes than the control group at the indicated concentrations (Figure 2C). However, the body weights did not significantly change in these experiments (Figure 2D). WST-1, caspase-3, and LDH activity assays were used to test the cytotoxic effects of PF at the indicated times (0, 8, 16, and 24 h; 20 μM) using the gastric cancer cell lines, SNU-638 and AGS. Based on Figure 2E–G, in the SNU-638 and AGS cells, the PF treatment reduced cell viability and enhanced LDH and caspase-3 activity in a time-dependent manner (0, 8, 16, and 24 h; 20 μM). Western blot analyses were performed to investigate the effect of the PF treatment on caspase-dependent apoptosis by PF treatment. The PF treatment significantly enhanced cleaved caspase-3 and caspase-9 cleavage in a time-dependent manner (0, 8, 16, and 24 h; 20 μM) (Figure 2H).

**Figure 3:**
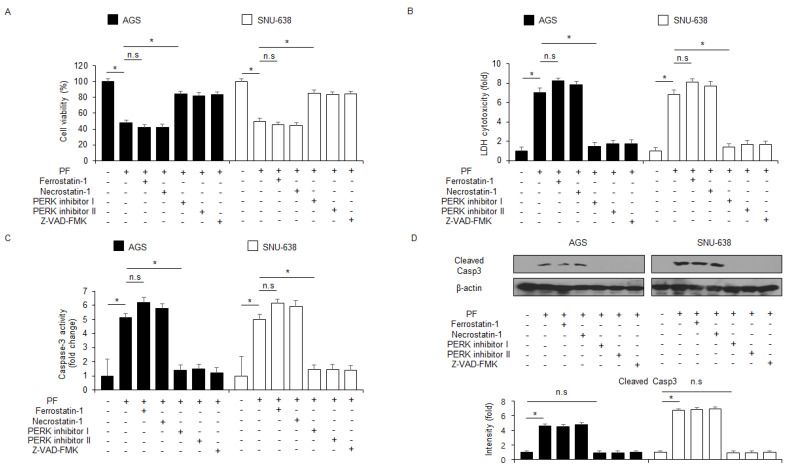
**PF mediates apoptotic cell death in gastric cancer cell lines.** (**A**–**D**) The effects of the necroptosis inhibitor necrostatin-1, the ferroptosis inhibitor ferrostatin-1, ER stress inhibitors PERK inhibitors I and II, and the pan-caspase inhibitor Z-VAD-FMK (50 mM; 24 h) on PF-induced apoptotic cell death. AGS and SNU-638 cells were pretreated with ferrostatin-1 (2 μM; 24 h), necrostatin-1 (20 μM; 24 h), PERK inhibitor I (10 μM; 24 h), PERK inhibitor II (10 μM; 24 h), or Z-VAD-FMK (50 mM; 24 h) and then treated with PF (20 µM; 24 h). WST-1, LDH, and caspase-3 activity assays were performed (*, *p* < 0.05, n.s = no significant). Total lysates were subjected to a western blot assay to identify the apoptosis marker caspase-3 cleavage. β-actin was used as the protein loading control.

To identify further if the PF effect is regulated by a pan-caspase inhibitor (Z-VAD-FMK), the cells were treated with PF (20 μM, 24 h) and Z-VAD-FMK (50 µM, 24 h). These findings indicated that Z-VAD-FMK inhibited the decrease of cell viability and reduced caspase-3 and LDH activity in PF-treated gastric cancer cells (Figure 3A–C). The western blot analyses showed that the Z-VAD-FMK and PF combined treatment reduced PF-mediated caspase-3 cleavage (Figure 3D).

### 3.3. PF Mediates Apoptotic Cell Death through ER Stress Pathway in Gastric Cancer Cells

**Figure 4:**
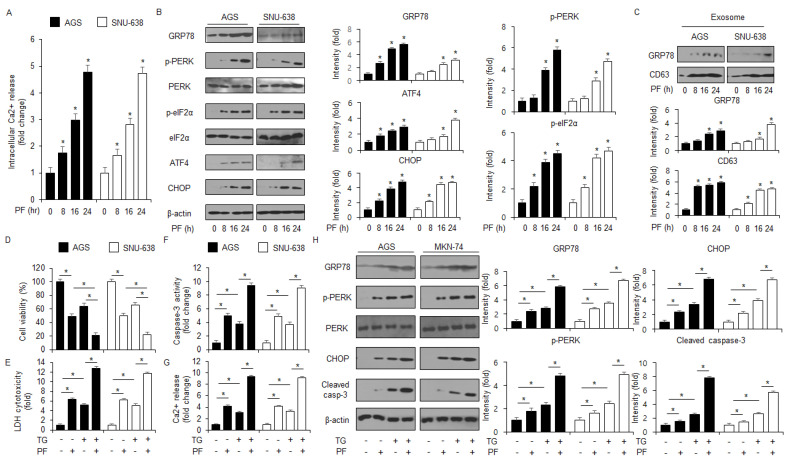
**PF mediates ER stress-caused apoptosis in gastric cancer cell lines.** (**A**) AGS and SNU-638 cells were treated with PF (20 μM) at the indicated times, and an intracellular Ca^2+^ assay was performed (* = *p* < 0.05, n.s = no significant). (**B**) The expression levels of eIF2α, p-PERK, CHOP, GRP78, ATF4, p-eIF2α, and PERK were measured using a western blot assay. β-actin was used as the protein loading control. (**C**) Gastric cancer cell lines were treated with PF (20 μM) at the indicated conditions. Exosomes (30 μg) were extracted from the cell culture medium. The exosome marker CD63 and the ER stress marker GRP78 were measured using a western blot assay on the exosome fractions extracted from the PF-induced gastric cancer cell culture medium. (**D**–**H**) Intracellular Ca^2+^, caspase-3 activity, LDH cytotoxicity, and WST-1 assays were performed, and the levels of the ER stress markers cleaved caspase-3, p-PERK, CHOP, PERK, and GRP78 were measured in the thapsigargin (TG; 3 μM; 24 h) and PF (20 μM; 24 h)-induced AGS and SNU-638 cell lines (* = *p* < 0.05, n.s = no significant). β-actin was used as the protein loading control. These experiments were repeated three times.

Certain studies have shown that ER stress responses by unfolded protein response (UPR) induce apoptosis and cell death in various cancer cell types [40,41]. To confirm whether PF regulates the ER stress response via the UPR in the gastric cancer cell lines AGS and SNU-638, an intracellular Ca^2+^ release assay was performed. The results of the assay suggest that PF time-dependently induces the intracellular Ca^2+^ release (Figure 4A). To confirm the expression levels of the essential ER stress proteins eIF2α, PERK, CHOP, p-eIF2α, p-PERK, ATF4, and GRP78 at the indicated times, western blot analyses were performed. The PF treatment up-regulated the protein expression levels of GRP78, and then it phosphorylated eIF2α and PERK (Figure 4B). In addition, these mediated the upregulation of ATF4 and CHOP levels (Figure 4B). To identify the role of GRP78-containing exosomes released from PF-treated gastric cancer cells, a western blot analysis was performed using the exosome fractions derived from the cell culture media. The PF treatment increased the expression of CD63 (the exosome marker) at the indicated times, and then GRP78 expression was dramatically higher compared with the control (Figure 4C). These results indicate that GRP78-containing exosomes might play a potential role in PF-mediated ER stress responses. Thapsigargin (TG) is an inhibitor of sarco/endoplasmic reticulum calcium ATPase (SERCA) and an inducer of ER stress response through disturbance of intracellular Ca^2+^ stores [32]. To investigate further whether PF regulates apoptotic cell death via the ER stress response in gastric cancer cells, the combinatory effect of TG and PF was investigated. These experiments showed that the combination of TG and PF reduced cell viability and enhanced LDH cytotoxicity, caspase-3 activity, and intracellular Ca^2+^ release compared with the control (Figure 4D–G). A western blot analysis showed that the combined treatment of PF and TG upregulated CHOP and cleaved caspase-3, GRP78, and p-PERK levels (Figure 4H).

### 3.4. Targeting the ER Stress Proteins Suppresses Apoptosis and Cell Death in PF-Treated Gastric Cancer Cell Lines

**Figure 5:**
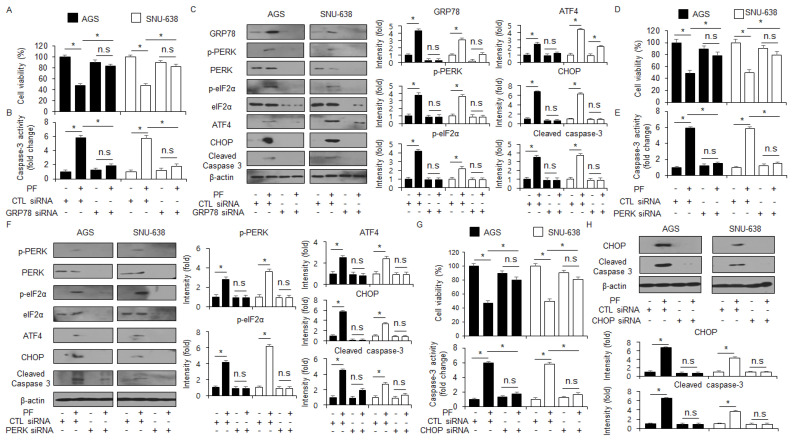
**Loss of GRP78, PERK, and CHOP suppresses apoptosis in PF-induced gastric cancer cell lines.** (**A**–**C**) GRP78 siRNA was transfected into gastric cancer cell lines following PF (20 μM; 24 h) treatment. Caspase-3 activity and WST-1 assays were performed, and the levels of the ER stress markers cleaved caspase-3, p-eIF2α, ATF4, p-PERK, CHOP, PERK, and GRP78 were measured (* = *p* < 0.05, n.s = no significant). β-actin was used as the protein loading control. These experiments were repeated three times. (**D**–**F**) PERK siRNA was transfected into gastric cancer cell lines following PF (20 μM; 24 h) treatment. Caspase-3 activity and cell viability assays were performed, and the levels of the ER stress markers PERK, CHOP, p-eIF2α, cleaved caspase-3, eIF2α, p-PERK, and ATF4 were measured (* = *p* < 0.05, n.s = no significant). β-actin was used as the protein loading control. These experiments were repeated three times. (**G**,**H**) CHOP siRNA was transfected into gastric cancer cell lines following PF (20 μM; 24 h) treatment. Caspase-3 activity and cell viability assays were performed, and the levels of cleaved caspase-3 and CHOP were measured (* = *p* < 0.05, n.s = no significant). β-actin was used as the protein loading control. These experiments were repeated three times.

To identify whether GRP78 is important in the apoptosis and cell death in PF-induced gastric cancer cells, a loss of function experiment targeting GRP78 was performed. The knockdown of GRP78 by RNA interference in the PF-treated AGS and SNU-638 cells suppressed the reduction in cell viability and the increase in caspase-3 activity in PF-mediated AGS and SNU-638 cells compared with the control cells (Figure 5A,B). Compared with the control, the PF treatment mediated the suppression of cleaved caspase-3, CHOP, p-eIF2α, ATF4, GRP78, and p-PERK expressions in the GRP78 knockdown AGS and SNU-638 cells. (Figure 5C). To investigate if targeting PERK signaling can alter cell fates, such as cell survival and cell death in cancer cells, a PERK knockdown experiment using PERK siRNA (30 nM; 24 h) was performed. PERK siRNA was transfected into the gastric cancer cell lines, such as AGS and SNU-638, were transfected with following the PF treatment. In the gastric cancer cell lines AGS and SNU-638, the PF treatment decreased cell viability and enhanced caspase-3 activity; however, with the knockdown of PERK, the PF treatment did not reduce cell viability or the increase of caspase-3 activity (Figure 5D,E). A western blot analysis showed that the PF treatment upregulated the levels of CHOP, p-PERK, ATF4, p-eIF2α, and cleaved caspase-3, compared with the control groups; however, the PERK knockdown groups did not show an increase in the levels of CHOP, cleaved caspase-3, and p-PERK in PF-induced gastric cancer cell lines such as AGS and SNU-638 (Figure 5F). To investigate further whether PF regulates the ER stress response in gastric cancer cells, a CHOP knockdown experiment was performed using CHOP siRNA (30 nM, 24 h). The PF treatment reduced cell viability and increased caspase-3 activity in the control cells, whereas the PF-treated CHOP knockdown cells had higher cell viability and less caspase-3 activity than the control groups (Figure 5G). In the western blot analysis, the control group had an upregulation in CHOP and caspase-3 cleavage in the PF-mediated gastric cancer cells; however, the knockdown of CHOP blocked the upregulation in CHOP and caspase-3 cleavage by the PF treatment (Figure 5H).

### 3.5. PF Induces Apoptosis by Generating ROS and ER Stress in Gastric Cancer Cell Lines

**Figure 6:**
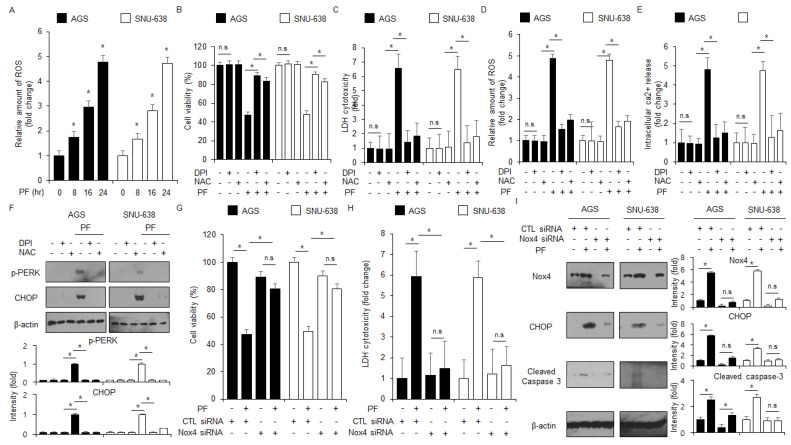
**ROS regulates apoptotic cell death and ER stress in PF-mediated gastric cancer cell lines.** (**A**) The fluorescence experiment indicates ROS generation using DCFDA in PF (0, 8, 16, and 24 h; 20 μM)-treated gastric cancer cells (*, *p* < 0.05, n.s = no significant). (**B**–**F**) AGS and SNU-638 cells were pretreated with NAC (100 μM) and DPI (1 μM) following PF (20 μM; 24 h) treatment. LDH activity, WST-1, and intracellular ROS and Ca^2+^ assays were performed (*, *p* < 0.05, n.s = no significant). (**F**) The expression levels of p-PERK and CHOP were measured using a western blot assay. β-actin was used as the protein loading control. These experiments were repeated three times. (**G**–**I**) Nox4 siRNA was transfected into gastric cancer cell lines following PF (20 μM; 24 h) treatment. LDH cytotoxicity and WST-1 assays were performed, and the levels of CHOP, caspase-3 cleavage, and Nox4 were measured (* = *p* < 0.05, n.s = no significant). β-actin was used as the protein loading control. These experiments were repeated three times.

To identify whether PF modulates ROS production in gastric cancer cell lines, a ROS detection experiment was performed. The PF treatment mediated the intracellular levels of ROS production at the indicated times (Figure 6A). To confirm whether NAC and DPI block the PF-induced apoptosis and ROS production in AGS and SNU-638 cells, intracellular Ca^2+^, ROS, LDH, and WST-1 assays were performed. The PF treatment, combined with NAC or DPI, suppressed the reduction of cell viability and the increase in LDH cytotoxicity, ROS release, and Ca^2+^ production to a greater extent than PF treatment alone (Figure 6B–E). In the western blot assay, the PF treatment in combination with NAC or DPI suppressed CHOP and p-PERK levels to a greater extent than the PF treatment alone groups (Figure 6F). These results suggest that PF induces apoptosis and ER stress by releasing ROS in gastric cancer cell lines.

### 3.6. PF Induces ER Stress-Mediated Apoptotic Cell Death via Nox4 in Gastric Cancer Cell Lines

To confirm if PF modulates Nox4-induced ROS generation, Nox4 siRNA was transfected into gastric cancer cell lines following the treatment with PF. The knockdown of Nox4 suppressed the reduction in cell viability and LDH release in the PF-treated gastric cancer cell lines AGS and SNU-638 compared with the control groups (Figure 6G,H). In the western blotting assay, the knockdown of Nox4 exerted a downregulation of Nox4, caspase-3 cleavage, and CHOP levels to a greater extent than the control groups (Figure 6I). These results suggest that Nox4 exerts ER stress-mediated apoptotic cell death by generating intracellular levels of ROS in PF-treated gastric cancer cells.

### 3.7. PF in Combination with Radiation Overcomes Radioresistance by Regulating EMT Events in Radio-Resistant Gastric Cancer Cells

**Figure 7:**
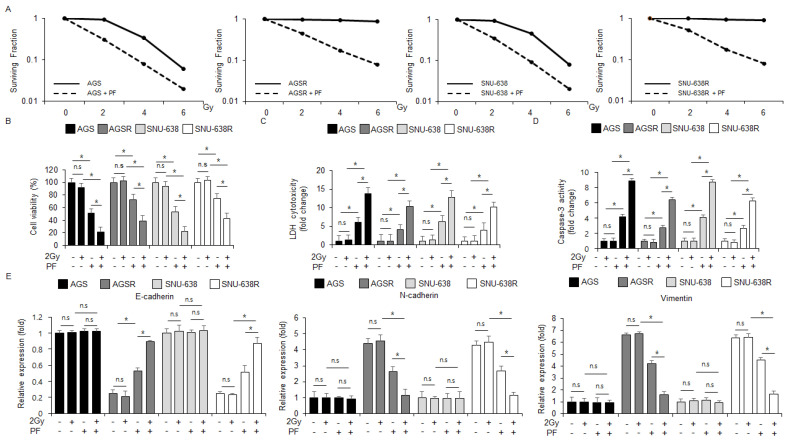
**PF combined with radiation (2 Gy) inhibits the EMT phenotype in radio-resistant gastric cancer cells.** (**A**) A colony formation assay was performed using AGS, SNU-638, AGSR, and SNU-638R cells treated with PF (20 μΜ; 24 h) indicated radiation condition doses (0, 2, 4, or 6 Gy). The clonogenic survival data were measured using the clonogenic fraction formula (* = *p* < 0.05, n.s = no significant). (**B**–**D**) LDH cytotoxicity, caspase-3 activity, and WST-1 assays were performed using radiation and PF-induced gastric cancer cell lines and radio-resistant gastric cancer cell lines (* = *p* < 0.05, n.s = no significant). (**E**) The mRNA levels of vimentin, E-cadherin, and N-cadherin were measured by qRT-PCR (* = *p* < 0.05, n.s = no significant). β-actin was used as the protein loading control. These experiments were repeated three times.

Radiation therapy is generally used for gastric cancer therapy, but for some gastric cancer patients, there are often difficulties acquiring radioresistance due to the promotion of epithelial-mesenchymal transition (EMT) [42]. To test if PF treatment can overcome radioresistance in the radio-resistant gastric cancer cells AGSR and SNU-638R, a colony formation assay was performed. The PF treatment exerted lower surviving fraction values at the indicated conditions (2, 4, and 6 Gy) in AGS, AGSR, SNU-638, and SNU-638R cells to a greater extent than the control groups (Figure 7A). In the AGS and SNU-638 cells, the PF treatment reduced cell viability and increased the caspase-3 activity and LDH cytotoxicity; the PF treatment combined with radiation (2 Gy) reduced cell viability and increased caspase-3 activity and LDH cytotoxicity even further, and radiation (2 Gy) alone had no effects on cell viability, caspase-3 activity and LDH cytotoxicity (Figure 7B–D). In the AGSR and SNU-638R cells, the PF treatment reduced cell viability and increased LDH and caspase-3 activity; the PF treatment combined with radiation (2 Gy) reduced cell viability and increased LDH and caspase-3 activity even further, and the effect of radiation (2 Gy) alone had no effects on cell viability, caspase-3 activity and LDH cytotoxicity (Figure 7B–D). To confirm if the PF treatment combined with PF and radiation (2 Gy) modulates EMT events in the radio-resistant gastric cancer cell lines SNU-638R, SNU-638, AGSR, and AGS cells, a quantitative real-time polymerase chain reaction (qRT-PCR) was performed. The qRT-PCR showed that the PF treatment and the PF treatment combined with radiation (2 Gy) downregulated the mRNA levels of N-cadherin and vimentin and up-regulated the mRNA levels of E-cadherin in AGSR and SNU-638R cells; however, the mRNA levels of E-cadherin, vimentin, and N-cadherin did not differ significantly in the AGS and SNU-638 cell lines (Figure 7E). Additionally, the mRNA levels of N-cadherin and vimentin were downregulated, and the mRNA levels of E-cadherin were upregulated in the PF-treated radiation (2 Gy)-induced AGSR and SNU-638R cell lines (Figure 7E). These results suggest that radiation in combination with the PF treatment could be a novel, powerful tumor therapeutic strategy to overcome radioresistance through the regulation of EMT events in AGSR and SNU-638R cell lines.

## 4. Discussion

In the present study, PF treatments inhibited inflammatory markers such as TNF-α, IL-6, and IL-1β in LPS-treated Raw264.7 cells and induced apoptotic cell death by activating the ER stress signaling pathway and releasing intracellular ROS in gastric cancer cells. Additionally, the PF treatment combined with 2 Gy overcame radioresistance via the activation of ER stress-mediated apoptotic cell death in radio-resistant SNU638R and AGSR cells.

In addition to the anti-inflammatory effects of PF treatments by the inhibition of IL-1β, IL-6, and TNF-α on LPS-treated Raw264.7 cells, the current study shows that PF treatments decreased tumor volume and induced cytotoxicity in gastric cancer cells in vivo and in vitro. This study also identified that PF treatments induce apoptosis by inducing the ER stress responses, including the PERK-ATF4-CHOP cascade, in gastric cancer cells. In addition, this study identified that radiation combined with PF treatments overcomes radioresistance and mediates apoptosis and cell death via the regulation of EMT makers, such as E-cadherin, N-cadherin, and vimentin, in gastric cancer cells and radiation-resistant gastric cancer cells.

Previous studies have shown that natural products and compounds can inhibit pro-inflammatory activators, including TNF-α, IL-6, and IL-1β [43]. Inflammation regulates cancer development, tumor growth, all stages of tumorigenesis, tumor formation, and metastasis in the tumor microenvironment [44]. Secreted inflammatory cytokines in cancer cells, including IL-22, IL-6, IL-8, IL-4, IL-1β, HGF, IGF-1, EGF, G-CSF, TNF-α, VEGF, and IL-11, can confer the promotion of chemo- and radioresistance [45]. Previous studies have reported the anti-inflammatory properties of natural products with minimal side effects and have suggested therapeutic ways to optimize the therapeutic efficacy of cancer therapies [46]. Many immunomodulatory natural products, such as flavonoids, flavones, phenolic compounds, and alkaloids, induce powerful anti-cancer efficacies via the modulation of NF-kB, iNOS, NO, Cox-2, and TNF-α [47]. It has been shown, in vitro and in vivo, that 2-himachelen-7-ol extracted from *Cedrus libani* exerts potential anti-cancer effects on various cancers, such as ovarian, colorectal, and brain. In addition, 2-himachelen-7-ol has been shown to mediate immunomodulatory effects by inhibiting induced Cox-2 expression in LPS-treated immune and cancer cells [48]. The flavonoid dihydroquercetin, extracted from onions, promotes anti-cancer and anti-inflammatory effects via the induction of apoptosis in cancer cells and the inhibition of pro-inflammatory agents, including NF-kB, TNFα, IL-1β, and IL-6, in LPS-treated cancer and immune cells [49]. With the findings of this study, the LPS treatment upregulated the expression levels of the cytokines TNFα, IL-1β, and IL-6 in Raw264.7 cells, whereas the combination treatment of PF and LPS downregulated TNFα, IL-1β, and IL-6 levels in Raw264.7 cells. These findings suggest that PF exerts a potential anti-inflammatory effect on immune cells.

Previous studies have indicated that PF induces apoptosis and cell death by regulating the caspase-dependent pathway in many cancers, such as gastric, oral, colon, liver, leukemia, and breast, and that it may also be a potential anti-cancer agent [50,51,52,53,54,55]. PF mediates anti-cancer effects by activating sirtuin 4, and it also overcomes tamoxifen resistance in estrogen receptor-positive breast cancer [47]. In the colorectal cancer cell lines LOVO and SW480, PF increased caspase-dependent apoptotic cell death by regulating invasion, migration, proliferation, and colony formation and by inhibiting the TLR4/NF-kB axis and EGFL7 [52]. In PF-treated hepatocellular cancer cells, HepG2 and SMMC-7721, PF mediated apoptosis by decreasing Wnt/β-catenin-related proteins, including C-myc, Cyclin-D1, and β-catenin [56]. PF-induced caspase-dependent apoptotic cell death via the reduction of cell viability, migration, and invasion, cell cycle arrest at the G2/M phase, the downregulation of NEDD4, and increased apoptosis detected using an annexin-V FACs assay [57]. In the colorectal cancer cell lines HCT116, PF exerted anti-cancer effects via inhibiting cell growth and FoxM1 and increasing annexin-V-stained cells, cell cycle arrest at the G0/G1 phase, and the expression of p21 and p27 [58]. In addition, PF mediated caspase-dependent apoptotic cell death by increasing annexin-V-stained cells, measured by flow cytometry, and the expression of Bax and caspase-3 cleavage and downregulating Bcl-2 in the human cervical cancer cell lines HeLa [59]. However, the molecular mechanism of the anti-cancer effects of PF treatment in gastric cancer is still not clear. This study shows that PF exerts a potential anti-cancer effect by inhibiting cell viability and tumor volume and inducing LDH and caspase-3 activity in vivo and in vitro in gastric cancer cell lines. In the western blot assays, PF-induced apoptosis via caspase-9 and caspase-3 cleavage and Z-VAD-FMK blocked the inhibition of cell viability, the induction of LDH and caspase-3 activity, and caspase-3 cleavage in PF-treated AGS and SNU-638 gastric cancer cells. These results suggest that PF exerts powerful anti-cancer effects through caspase-dependent apoptotic cell death in vivo and in vitro in gastric cancer.

Previous studies have shown that ER stress response activates the UPR and induces apoptosis and cell death in various cancer cell types [60,61]. ER stress plays a crucial role in various cellular environments and regulates cell survival and cell death [62]. The ER lumen is important in post-translational modification, protein folding of secretory proteins, and maintaining calcium and redox conditions for cellular homeostasis. However, if this process fails, the accumulation of unfolded proteins initiates ER stress via the activation of ER transmembrane proteins, such as PERK, IRE1α, and ATF6 [63]. Mild ER stress contributes to cell homeostasis via the activation of signaling pathways for cell survival [62]. However, strong or accumulated ER stress induces apoptotic cell death via the activation of the CHOP-DR4/5-caspase-8/9-caspase-3 axis in various cancer cell lines, indicating a potential strategy for tumor therapy [60,64]. In addition, an anti-cancer effector of the immune cancer environment is the differentiation into CD8^+^ T cells (cytotoxic T lymphocytes) that kill cancer cells; ROS induces immunogenic cell death by activating ER stress [65]. This study also indicated that PF induces apoptotic cell death by activating ER stress-mediated pathways in gastric cancer cell lines. Many ER stress-related molecules, such as Bcl-2/Bcl-X_L_, microRNAs, calcium, MEKK1, kinase regulatory pathways, and transcription factors, induce apoptotic cell death by activating caspases [66]. This study indicated that PF induces caspase-3-dependent apoptotic cell death in AGS and SNU-638 cells. To investigate further whether PF mediates caspase signaling pathway-dependent apoptotic cell death, a pharmacological experiment was performed using the caspase inhibitor Z-VAD-FMK. PF combined with Z-VAD-FMK blocked PF-induced apoptosis in gastric cancer cells. These results suggest that PF induces cell death and apoptosis via a caspase-dependent axis in gastric cancer. Next, to determine whether PF combined with TG regulates apoptosis, a combination therapy was performed. The combined treatment of PF and TG induced higher apoptotic cell death via the activation of caspase-3 and ER stress response-related markers compared with PF treatment alone. At the beginning of the ER stress response, the dissociation between GRP78 and PERK induces the autophosphorylation of PERK, which activates PERK [67]. The phosphorylation of PERK phosphorylates of eIF2α in the cytosol [68,69]. Targeting PERK signaling can alter cell fates, such as cell survival and cell death in cancer cells [70]. eIF2α activates ATF4 in the cytosol, and then ATF4 is translocated to the nucleus [71]. Nuclear ATF4 interacts with the promoter of the transcription factor CHOP. The induction of CHOP induces apoptotic cell death in response to ER stress [72]. This study shows that the PF treatment induces the upregulation of GRP78, ATF4, and CHOP, as well as the phosphorylation of PERK and eIF2α via the release of intracellular Ca^2+^ in a time-dependent manner in gastric cancer cells. However, the knockdown of the ER stress proteins CHOP, GRP78, and PERK suppressed cell death and apoptosis by inhibiting caspase-3 activity and downregulating caspase-3 cleavage, CHOP, p-PERK, and GRP78 in PF-treated gastric cancer cells. Recent reports have shown that exosomes derived from cancer cells induce apoptosis via the activation of ER stress in cancer [73,74]. Recent studies have reported that GRP78-containing exosomes released in response to ER stress mediate cell-to-cell communication, cell survival, and cell death in cancer [75]. The exosome fraction had increased expression levels of GRP78 in a time-dependent manner in PF-treated AGS and SNU-638 gastric cancer cells. These results suggest that GRP78-containing exosomes might play a potential role in the PF-mediated ER stress response.

Previous studies have shown that the crosstalk between redox and ER stress is an important modulator for cellular processes to decide cell fate [76]. Oxidative stress causes the imbalance of cellular redox homeostasis through the generation of ROS [77]. The major factors in ROS production are protein disulfide isomerase (PDI), the NADPH oxidase complex (Nox2 and Nox4), ER oxidoreductin (ERO-1), and calcium. These factors are potential regulators of the ER stress response [78]. These findings suggest that PF regulates intracellular ROS production, calcium release, and the ER stress response by increasing NOX4 activity in the gastric cancer cell lines AGS and SNU-638. In addition, the loss of function experiments using Nox4-specific siRNA or DPI (Nox4 inhibitor) and NAC (ROS inhibitor) suppressed cell death, apoptosis, and the ER stress-mediated PERK axis in PF-treated AGS and SNU-638 cells.

In cancer therapies, radiation therapy, also named radiotherapy, is a treatment to kill cancer cells using radiation; however, it frequently causes radioresistance [79]. To overcome radioresistance and improve the effect of radiotherapy in cancer, there is a need to develop detailed molecular mechanisms, possible therapeutic targets, and novel combined therapies. The natural product resveratrol has a potential anti-cancer effect via ROS generation in radiation-exposed non-small cell lung cancer (NSCLC) [80]. Resveratrol combined with radiation exerts a synergistic anti-prostate cancer effect by reducing tumor growth in xenograft mouse models [81]. In addition, the natural product curcumin combined with radiation induces a powerful anti-cancer effect in colon cancer models [82]. Previous studies have shown that radiation combined with the natural product nodakenin overcomes radioresistance by activating ROS, ER stress, and apoptosis in vivo and in vitro in radio-resistant breast cancer models [83]. Radiation combined with the natural product saikosaponin A overcomes radioresistance via the induction of apoptotic cell death in vitro and in vivo in radio-resistant gastric cancer models [84]. The natural flavonoid fisetin combined with radiation also overcomes radioresistance and has a powerful anti-cancer effect in vitro and in vivo in radio-resistant liver cancer models [85]. These results suggest that PF combined with radiation exerts a more effective anti-cancer effect and overcomes radioresistance by regulating EMT markers, such as E-cadherin, N-cadherin, and vimentin, in radio-resistant gastric cancer cells. In addition, PF combined with radiation mediates the synergistic inhibition of cell viability and activates LDH and caspase-3 activity in gastric cancer cells and radio-resistant gastric cancer cells.

## 5. Conclusions

This study indicates that PF, an anti-inflammatory reagent, blocks cancer cell proliferation and growth by regulating cell death, apoptosis, and Nox4-mediated ER stress response in gastric cancer cells in vivo and in vitro. These findings contribute to the biological concept and insight into the detailed molecular pathways of PF as a gastric tumor therapeutic strategy. In addition, these results create a novel bridge between radiation and natural compounds in cancer radiotherapy and suggest a novel therapeutic strategy and approach for cancer therapies.

## Data Availability

The data are contained within the article.
